# Sestrin2 Regulates Endoplasmic Reticulum Stress-Dependent Ferroptosis to Engage Pulmonary Fibrosis by Nuclear Factor Erythroid 2-Related Factor 2/Activating Transcription Factor 4 (NRF2/ATF4)

**DOI:** 10.1155/2023/9439536

**Published:** 2023-11-07

**Authors:** Zhaoxing Dong, Ting Li, Cenli Wang, Yong Zhou, Zhongkai Tong, Xuekui Du

**Affiliations:** ^1^Department of Respiratory and Critical Care Medicine, Ningbo No. 2 Hospital, Ningbo 315000, Zhejiang Province, China; ^2^Department of Respiratory and Critical Care Medicine, Xiangshan Red Cross Taiwan Compatriot Hospital Medical and Health Group, Ningbo 315000, Zhejiang, China

## Abstract

Pulmonary fibrosis (PF) can lead to chronic inflammation, the destruction of alveoli and irreversible lung damage. Sestrin2 is a highly protective stress-inducible protein that is involved in the cell response to various stress factors and the regulation of homeostasis and has a certain protective effect against PF. In this study, TGF-*β*1 was used to establish a PF cell model. Bleomycin was used to induce PF in mice, and the expression levels of related proteins were detected by western blotting. The levels of the inflammatory cytokine, TNF-*α*, IL-6, and IL-1*β* were detected by enzyme-linked immunosorbent assays. Immunoprecipitation was used to verify the interaction between ATF4 and NRF2 and between Sestrin2 and NRF2 to explore the specific mechanism by which Sestrin2 affects PF. The results showed that Sestrin2 inhibited fibroblast-to-myofibroblast transition (FMT), improved inflammation, promoted cell proliferation, and alleviated PF. Activating transcription factor 4/nuclear factor erythroid 2-related factor 2 (NRF2/ATF4) signaling pathway activation could alleviate endoplasmic reticulum stress, inhibit ferroptosis and FMT, and reduce reactive oxygen species levels, thereby alleviating PF. Overexpression of ATF4 and the addition of a ferroptosis inducer reversed Sestrin2-mediated alleviation of PF. In conclusion, Sestrin2 alleviates PF and endoplasmic reticulum stress-dependent ferroptosis through the NRF2/ATF4 pathway.

## 1. Introduction

Pulmonary fibrosis (PF) is a progressive lung disease that results from abnormal scarring following repeated injury to alveolar epithelial or endothelial cells, thereby triggering chronic inflammation and the sedimentation of extracellular matrix proteins and leading to fibrotic reconstruction and irreversible damage to alveoli and lung function [1]. Idiopathic pulmonary fibrosis (IPF) is one of the most common types of PF, its cause is unknown, and it has high-mortality rate and limited treatment options [2]. PF can be caused by multiple factors, including genetic defects, autoimmune diseases, and environmental exposure. Recent studies suggest that immune dysregulation may be a key driver of PF [3, 4]. Often, PF is progressive, which, combined with the heterogeneous nature of the disease, makes treatment increasingly difficult [5, 6]. Therefore, it is important to investigate molecules that effectively improve PF and their regulatory mechanisms to improve the treatment of lung fibrosis.

Sestrin2 is a member of a highly conserved class of stress-inducible proteins that plays a significant role in cellular stress states. Sestrin2 can be induced by a variety of stress factors, including DNA lesions, oxidative stress, endoplasmic reticulum (ER) stress, and metabolic stress [7, 8]. In our previous report, Sestrin2 was shown to inhibit cell proliferation during lung fibrosis, promote apoptosis, and activate autophagy through a ceRNA mechanism, thereby alleviating the progression of lung fibrosis [9]. In addition, Sestrin2 was shown to exert cytoprotective effects by reducing endoplasmic reticulum stress [10–12]. Ding et al. [13] showed that under ER stress induced by glucose starvation, Sestrin2 upregulation was dependent on NRF2/ATF4 signaling pathway activation, thereby protecting cells from nonprogrammed necrosis. Thus, the modulation of Sestrin2 activity can alleviate PF, and exploring the molecular mechanisms by which Sestrin2 regulates PF can provide new therapeutic targets.

Ferroptosis is a unique form of iron-dependent cell death. Mitochondria-induced cysteine deprivation, endoplasmic reticulum-associated oxidative stress, lysosomal dysfunction, and Golgi stress-related lipid peroxidation have been reported to be conducive to the induction of ferroptosis [14]. Among them, endoplasmic reticulum stress is closely related to the onset and development of fibrotic diseases, and Burman et al. [15] showed that ER stress induced reactive oxygen species (ROS) accumulation and played a key pathogenic role in PF. Numerous studies have shown that ferroptosis plays a crucial role in the development of PF [16–18] and that ferroptosis causes intracellular oxidative stress and inflammation, resulting in an increase in intracellular ROS levels and ultimately promoting the progression of PF. In addition, ferroptosis can lead to different immune and inflammatory reactions through the release and activation of different damage-associated molecular pattern (DAMP) signals, which further exacerbates lung fibrosis [19]. These studies confirmed that endoplasmic reticulum stress induces ROS production, which exacerbates lipid peroxidation and promotes ferroptosis, suggesting that PF can be alleviated by inhibiting ferroptosis.

The activation of ATF4, a molecular component of the endoplasmic reticulum stress pathway, can promote endoplasmic reticulum stress and induce ferroptosis [20]. Studies have identified signaling pathways that are key regulators of cellular ferroptosis, and among those signaling pathways, the NRF2/heme oxygenase-1 (HO-1) signaling pathway strictly regulates ferroptosis [21]. ATF4 has been reported to act as an NRF2-interacting protein to induce HO-1 expression and thus exert cytoprotective effects [22, 23]. During endoplasmic reticulum stress, NRF2 controls the gene expression of endogenous antioxidant components and ROS-clearing enzymes, and ATF4-dependent NRF2 transfer and regulation accelerates antioxidant defense [24].

Thus, this study examined the specific mechanism by which Sestrin2 regulates endoplasmic reticulum stress-dependent ferroptosis through the NRF2/ATF4 pathway, which further affects PF and provides a new theoretical basis for the clinical treatment of PF.

## 2. Experimental Methods

### 2.1. Cell Culture

MRC-5 human embryonic lung fibroblasts (Cell Bank of the Chinese Academy of Sciences) were cultured in DMEM at 37°C in 5% CO_2_. The cells were transfected with Sestrin2, ATF4, and NRF2 overexpression plasmids or inhibitors (Oligobio, China) and negative control null plasmids when they reached approximately 90% confluence according to the instructions of Lipofectamine 3000 (Thermo Fisher Scientific, USA). Model cell groups were treated with 10 ng/mL TGF-*β*1 (Sigma, USA). An ER stress inhibitor (4-PBA, 0.3 mM) and a ferroptosis inducer (RSL3, 0.1 *µ*m) were used.

### 2.2. Cell Counting Kit-8 (CCK-8)

The viability of MRC-5 cells was measured by a CCK-8 kit (Solarbio, China). MRC-5 cells (4 × 10^3^) were inoculated into 96-well plates. After the cells reached confluence, the supernatant was discarded, and new medium was added. The cells were treated with different concentrations of TGF-*β*1 (0, 1, 2.5, 5, 10, and 20 ng/mL), and the plates were incubated at 37°C and 5% CO_2_ for 24 hr. Then, CCK-8 solution was added to the wells, and the enzyme label was modulated. The absorbance was measured at 450 nm.

### 2.3. Immunofluorescence Analysis

The cells were evaluated by immunofluorescence staining. The cells were washed with PBS, treated with 4% paraformaldehyde (Thermo Scientific) for 20 min, blocked with PBS containing 5% bovine serum protein for 1 hr, and then incubated with primary antibodies against CHOP or *α*-SMA (1 : 3,000, Cell Signaling Technology, USA). The cells were incubated with PerCP Cyanine 5.5-labeled goat anti-mouse antibodies (1 : 5,000, LI-COR Biosciences, USA) for 1 hr in the dark. The nuclei were stained with DAPI, and the cells were photographed by the fluorescence microscopy.

### 2.4. RT‒PCR

Total RNA was extracted using TRIzol reagent according to the manufacturer's instructions, and cDNA was synthesized using a Reverse Transcriptase Kit (TAKARA, USA). Then, qPCR was performed in a real-time system (Bio-Rad) by using a YBR Green PCR Kit (KM4101, KAPA Biosystems). Each reaction was performed in duplicate. The reaction procedure was as follows: predenaturation at 95°C for 3 min, 95°C for 5 s, 56°C for 10 s, and 72°C for 25 s (40 cycles). The results were analyzed using the 2^−△△Ct^ method. GAPDH was used as a reference to normalize the gene expression data. The primers were designed and configured by Sangon Biotechnology Co., Ltd. and are listed in Table 1.

### 2.5. Western Blot Analysis

Total proteins were extracted and quantified by the bicinchoninic acid assays. The proteins were separated using 10% SDS‒PAGE gels and transferred to PVDF membranes. The membranes were blocked with PBS buffer containing 5% nonfat milk and 0.05% Tween-20 for 2 hr, followed by incubation with primary antibodies (Abcam, UK) for 1 hr at room temperature. After three washes with PBS/Tween 20, the membranes were incubated with horseradish peroxidase-conjugated secondary goat anti-rabbit IgG (1 : 20,000, SAB43714, Bioswamp) for 2 hr at room temperature. Protein bands were visualized by enhanced chemiluminescence color detection, and ImageJ software was used for protein band analysis.

### 2.6. ROS Analysis

Each sample was incubated with 500 *μ*L of D-Hanks solution containing DCFH-DA (20 *μ*L) for 30 min in the dark, and the cells were shaken every 5 min to allow them to fully bind to the probe. The cells were resuspended in D-Hanks solution, and ROS levels were detected by the flow cytometry.

### 2.7. Fe^2+^ Detection

Fe^2+^ levels in cells were examined using an Iron Assay Kit (Sigma‒Aldrich, St. Louis, MO, USA) according to the manufacturer's instructions and as previously described by Ding et al. [25].

### 2.8. Measurement of MDA and SOD

The levels of SOD and MDA were evaluated using Assay Kits (Nanjing Jiancheng Bioengineering Institute) according to the manufacturer's instructions.

### 2.9. Enzyme-Linked Immunosorbent Assay (ELISA)

TNF-*α*, IL-6, and IL-1*β* levels in cell supernatants were quantified using ELISA kits (RiboBio, Guangzhou, China). The samples were added to the wells, biotin-labeled antibodies were added, and then the plate was incubated at 37°C for 2 hr. Then, horseradish peroxide-labeled streptavidin was added, and the plate was incubated for 1 hr. Finally, chromogenic solution was added and incubated for 30 min. The reaction was terminated, and the OD value was measured at 450 nm.

### 2.10. Bimolecular Fluorescence Complementation (BiFC)

Human NRF2 cDNA and ATF4 cDNA were inserted into the BiFC vectors pBiFC-VC155 and pBiFC-VN173 (Sangon Biotech, China). The recombinant plasmids were cotransfected into cells. After 24 hr, the cells were fixed with 4% paraformaldehyde, incubated with DAPI for 5 min, and observed and photographed under a confocal microscope (Nikon Instruments Inc.) at excitation and emission wavelengths of 488 and 500 nm, respectively.

### 2.11. Immunoprecipitation (IP)

Total protein was isolated from MRC-5 cells using total protein extraction buffer. Protein A/G Sepharose beads (Santa Cruz Biotechnology) were preincubated with anti-ATF4 and anti-Sestrin2 (1 : 50, CST) antibodies for 60 min at 4°C with slow shaking and then washed twice. All IP samples were incubated overnight with slow shaking, the beads were collected by centrifugation and then washed three times with lysis buffer. The immunoprecipitates were analyzed by western blotting.

### 2.12. Construction of a Mouse Lung Fibrosis Model

Experimental C57BL/6 mice (male, 4–6 weeks old, 18–20 g) were acquired from the Animal Experiment Center of Kunming Medical University. Then, the mice were anesthetized with 0.6% pentobarbital sodium, and 2.5 mg/kg bleomycin was injected into the trachea according to the method described by Wang et al. [26] to establish the PF model. NRF2 gene expression adenoviruses, ATF4 gene expression adenoviruses and their controls were purchased from GenePharma Technology Co., Ltd. One week before the experiment, 50 *µ*L of adenovirus was injected into the tail vein at a dose of 10 nmol/mouse. The transfection efficiency was determined by western blotting.

All animal experimental protocols were approved by the Animal Experiment Ethics Committee of Kunming Medical University (approval number: kmmu2021744), and the animal procedures adhered to the ARRIVE guidelines 2.0.

### 2.13. Immunohistochemical Staining

Paraffin sections were placed in the oven at 65°C for 30 min, dewaxed with xylene and dehydrated with alcohol. After heated antigen repair, endogenous enzyme activity was inactivated with 3% H_2_O_2_, and the samples were blocked with goat serum for 1 hr. The anti-Sestrin2 primary antibody (ab178518, 1/100, Abcam) was added and incubated overnight at 4°C. The HRP-labeled secondary antibody was added and incubated at 37°C for 30 min, and the samples were processed for DAB color development. The sections were counterstained with hematoxylin, sealed with neutral gum and observed under the microscope.

### 2.14. Masson Staining

Paraffin sections were dewaxed, hydrated in a graded ethanol series, stained with hematoxylin, and washed with water. Masson's trichrome red was used as an acidic reddish solution, and the sections were washed with 2% glacial acetic acid in an aqueous solution for one minute. The sections were differentiated with 1% phosphomolybdic acid in an aqueous solution without being washed with water, directly stained with aniline blue or light green solution for 5 min, and washed with 0.2% glacial acetic in an acid aqueous solution for 1 min. Then, the sections were dehydrated with 95% alcohol and anhydrous alcohol, made transparent with xylene, and a neutral adhesive sealing film was applied. The slides were observed and photographed with a microscope.

### 2.15. HematoxylinEosin (HE) Staining

Paraffin sections were dewaxed, hydrated in a graded ethanol series, stained with hematoxylin, subjected to acid water and ammonia color separation, rinsed with running water, decolorized in 70% and 90% ethanol in sequence, stained with alcohol eosin, and dried at room temperature. Xylene was used to establish transparency, and resin was used for sealing. The cells were observed under a microscope and photographed.

### 2.16. Statistical Analysis

The data are expressed as the mean ± standard deviation, and differences between groups were tested by one-way analysis of variance and Student's *t* test.

## 3. Results

### 3.1. Sestrin2 Overexpression Reduced ER Stress and Ferroptosis

**Figure 1:**
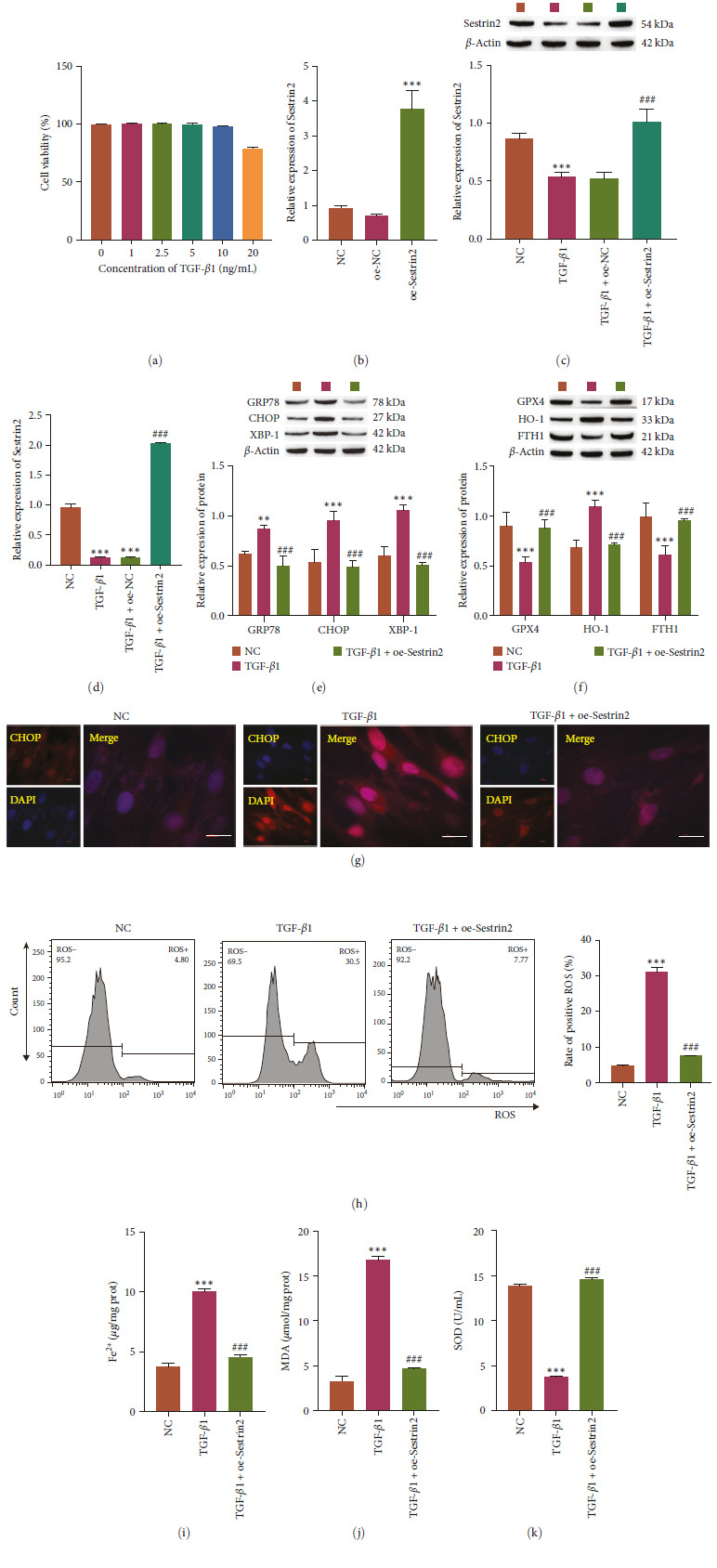
Sestrin2 overexpression reduces endoplasmic reticulum stress and ferroptosis levels. (a) CCK-8 assay of cell viability after treatment with different concentrations of TGF-*β*1 (0, 1, 2.5, 5, 10, and 20 ng/mL, *n* = 3). (b) RT‒qPCR of Sestrin2 expression (*n* = 3). (c) Western blot analysis of Sestrin2 expression (*n* = 3). (d) RT‒qPCR of Sestrin2 expression (*n* = 3). (e) Western blot analysis of ER stress-related protein expression (GRP78, CHOP and XBP-1, *n* = 3). (f) Western blot analysis of ferroptosis-related protein expression (GPX4, HO-1, and FTH1, *n* = 3). (g) Immunofluorescence analysis of CHOP localization (scale bar = 20 *µ*m, *n* = 3). (h) Flow cytometric analysis of ROS levels. (i) Analysis of Fe^2+^ levels (*n* = 3). (j and k) Oxidative stress factor analysis of MDA and SOD expression (*n* = 3) vs. NC,  ^*∗∗*^*P<*0.01,  ^*∗∗∗*^*P<*0.001 vs. TGF-*β*1, ^###^*P<*0.001.

MRC-5 cells were induced with TGF-*β*1, and endoplasmic reticulum stress and ferroptosis levels were detected. First, CCK-8 was used to detect cell viability in response to different concentrations of TGF-*β*1 to determine the optimal concentration of TGF-*β*1 (10 ng/mL) (Figure 1(a)). Sestrin2 expression was downregulated in MRC-5 cells induced by TGF-*β*1, and sestrin2 was successfully overexpressed in MRC-5 cells (Figure 1(b)–1(d)). In addition, in TGF-*β*1-induced MRC-5 cells, we found that GRP78, CHOP, XBP-1 and HO-1, expression levels were upregulated, GPX4 and FTH1 expression levels were downregulated, and Sestrin2 overexpression reversed these effects (Figure 1(e)–1(f)). The results of immunofluorescence analysis of CHOP were consistent with the western blot results (Figure 1(g)). TGF-*β*1 increased the levels of ROS and MDA, increased the accumulation of Fe^2+^ and decreased the expression of SOD. After Sestrin2 overexpression, the effect of TGF-*β*1 on MRC-5 cells was reversed (Figure 1(h)–1(k)). These results show that Sestrin2 overexpression can reduce endoplasmic reticulum stress and inhibit ferroptosis.

### 3.2. Sestrin2 Overexpression Inhibits the Transformation of Fibroblasts into Myofibroblasts and the Expression of Inflammatory Cytokines

**Figure 2:**
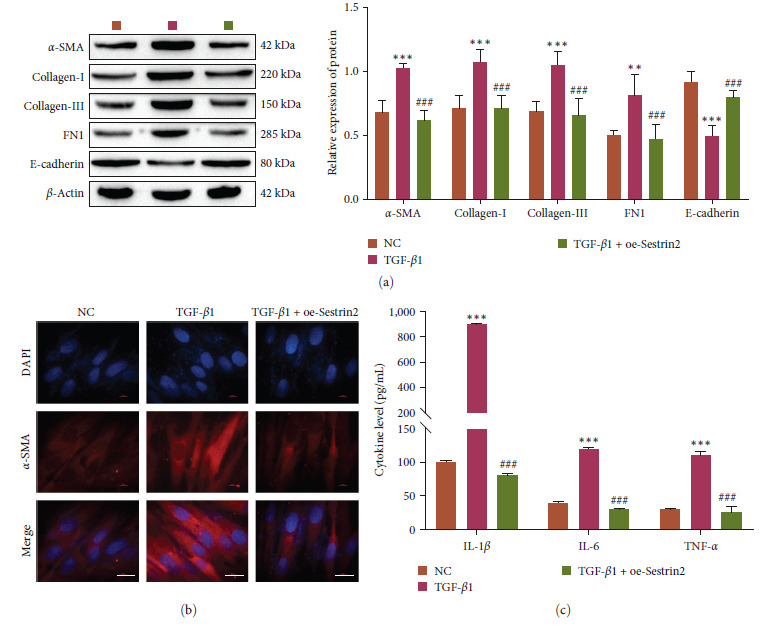
Sestrin2 overexpression reduces pulmonary fibrosis. (a) Western blot showing the expression of the FMT-related proteins *α*-SMA, collagen-I, collagen-III, FN1, and *E*-cadherin (*n* = 3). (b) Immunofluorescence analysis of the expression of *α*-SMA (scale bar = 20 *µ*m, *n* = 3). (c) ELISA analysis of the expression of the inflammatory factors IL-1*β*, IL-6, and TNF-*α* (*n* = 3) vs. NC,  ^*∗∗*^*P<*0.01,  ^*∗∗∗*^*P<*0.001 vs. TGF-*β*1, ^###^*P<*0.001.

To verify the effect of Sestrin2 on MRC-5 cells, the expression of proteins associated with fibroblast transformation into myofibroblasts (*α*-SMA, Collagen-I, Collagen-III, FN1, and E-cadherin) was detected by the western blotting and immunofluorescence analysis, and the expression of inflammatory factors (IL-1*β*, IL-6 and TNF-*α*) was detected by ELISA. The results showed that the expression levels of *α*-SMA, Collagen-I, Collagen-III, FN1, TNF-*α*, IL-6, and IL-1*β* were increased, and the expression of *E*-cadherin was decreased in the TGF-*β*1 group. Sestrin2 overexpression reversed the effect of TGF-*β*1 on these indicators (Figure 2). Sestrin2 overexpression can inhibit the transformation of fibroblasts into myofibroblasts and inhibit the expression of inflammatory factors.

### 3.3. Regulation of the NRF2/ATF4 Signaling Pathway Can Affect ER Stress-Dependent Ferroptosis Levels

**Figure 3:**
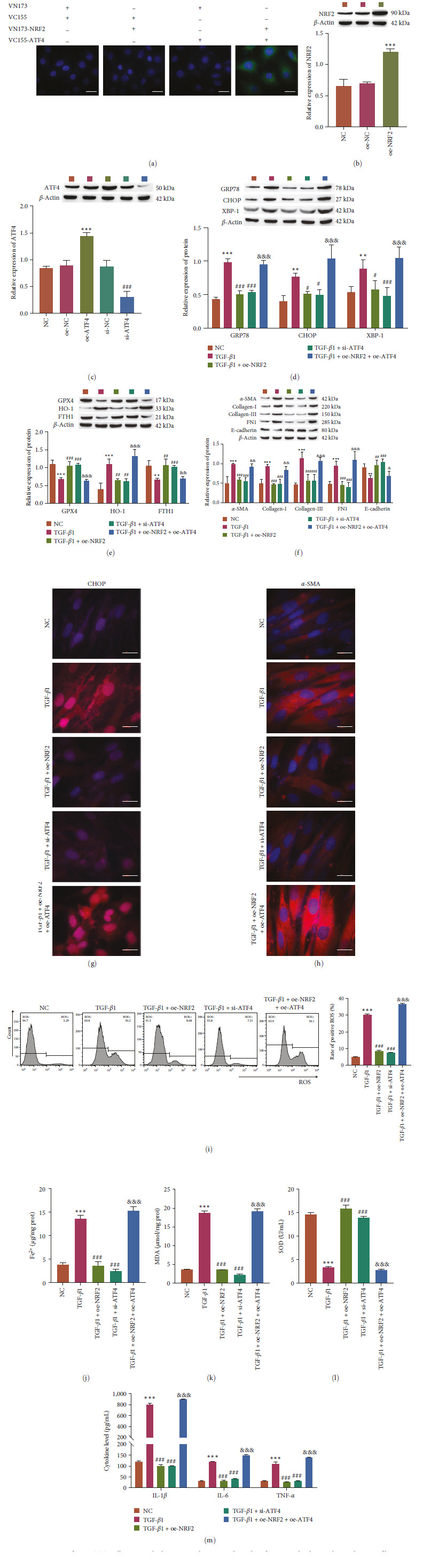
Activation of NRF2/ATF4 alleviates endoplasmic reticulum stress-dependent ferroptosis levels to reduce pulmonary fibrosis. (a) BiFC analysis of the interaction between NRF2 and ATF4 (scale bar = 20 *µ*m, *n* = 3). (b and c) Western blot analysis of the expression of ATF4 and NRF2 (*n* = 3). (d) Western blot analysis of the expression of ER stress-related proteins (GRP78, CHOP, and XBP-1, *n* = 3). (e) Western blot analysis of the expression of ferroptosis-related proteins (GPX4, HO-1, and FTH1, *n* = 3). (f) Western blot analysis of the expression of FMT-related proteins (*α*-SMA, collagen-I, collagen-III, FN1, and E-cadherin, *n* = 3). (g and h) Immunofluorescence analysis of CHOP and *α*-SMA expression (scale bar = 20 *µ*m, *n* = 3). (i) Flow cytometric analysis of ROS levels (*n* = 3). (j) Analysis of Fe^2+^ levels (*n* = 3). (k and l) Oxidative stress factor analysis to determine the expression of MDA and SOD (*n* = 3). (m) ELISA analysis of the expression of inflammation-related factors (IL-1*β*, IL-6, and TNF-*α*, *n* = 3) vs. NC,  ^*∗∗*^*P<*0.01,  ^*∗∗∗*^*P<*0.001 vs. TGF-*β*1, ^#^*P<*0.05, ^##^*P<*0.01, ^###^*P<*0.001 vs. TGF-*β*1 + oe-NRF2, ^&^*P<*0.05, ^&&^*P<*0.01, ^&&&^*P<*0.001.

We examined the effect of the NRF2/ATF4 pathway on a PF cell model. In MRC-5 cells, the activity of the NRF2/ATF4 signaling pathway was investigated after the overexpression of NRF2 and knockdown or overexpression of ATF4. Firstly, direct physical interaction of NRF2 with ATF4 was observed in the BiFC assay (Figure 3(a)), which is consistent with the previous reports [23]. Western blot analysis showed successful transfection of oe-NRF2, oe-ATF4, and si-ATF4 (Figure 3(b)–3(c)). Compared with that in the TGF-*β*1 group, overexpression of NRF2 or knockdown of ATF4 inhibited the expression of certain proteins (GRP78, CHOP, XBP-1, HO-1, *α*-SMA, Collagen-I, Collagen-III, and FN1) and promoted the expression of certain proteins (GPX4, FTH1, and E-cadherin). Compared with the TGF-*β*1 + oe-NRF2 group, overexpression of ATF4 increased the protein levels of GRP78, CHOP, XBP-1, HO-1, *α*-SMA, Collagen-I, Collagen-III, and FN1 and inhibited the protein levels of GPX4, FTH1 and *E*-cadherin (Figure 3(d)–3(f)). The results of immunofluorescence analysis of CHOP and *α*-SMA were consistent with the western blot results (Figures 3(g) and 3(h)). In addition, overexpression of NRF2 or knockdown of ATF4 inhibited the production of ROS and the accumulation of Fe^2+^ and MDA and promoted the expression of SOD compared with those in the TGF-*β*1 group. Compared with those in the TGF-*β*1 + oe-NRF2 group, overexpression of ATF4 reversed these effects (Figure 3(i)–3(l)). In addition, ELISA showed hat overexpression of NRF2 or knockdown of ATF4 decreased inflammatory factors (IL-1*β*, IL-6, and TNF-*α*) (Figure 3(m)). In conclusion, the NRF2/ATF4 signaling pathway can regulate ER stress-dependent ferroptosis.

### 3.4. Sestrin2 Regulates NRF2 to Influence the Level of Ferroptosis under ER Stress Conditions

**Figure 4:**
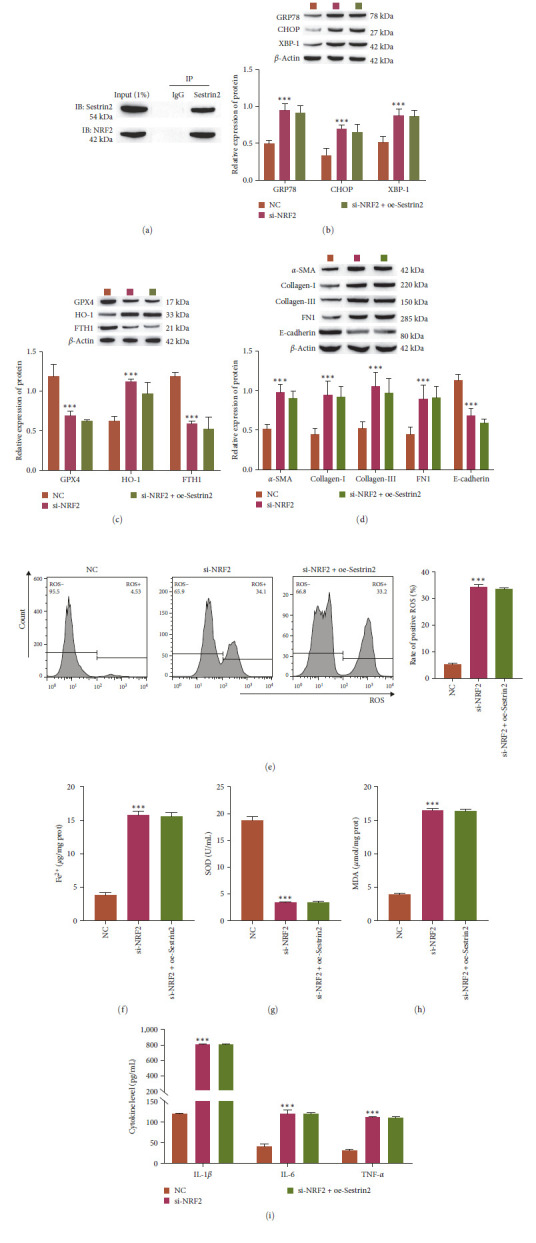
Sestrin2 attenuates endoplasmic reticulum stress-dependent ferroptosis through the NRF2/ATF4 pathway. (a) Immunoprecipitation was performed to verify the interaction between Sestrin2 and NRF2 (*n* = 3). (b) Western blot analysis of the expression of ER stress-related proteins (GRP78, CHOP, and XBP-1, *n* = 3). (c) Western blot analysis of the expression of ferroptosis-related proteins (GPX4, HO-1, and FTH1, *n* = 3). (d) Western blot analysis of the expression of FMT-related proteins (*α*-SMA, collagen-I, collagen-III, FN1, and *E*-cadherin, *n* = 3). (e) Flow cytometric analysis of ROS levels (*n* = 3). (f) Analysis of Fe^2+^ levels (*n* = 3). (g and h) Oxidative stress factor analysis to determine the expression of MDA and SOD (*n* = 3). (i) ELISA analysis of the expression of inflammation-related factors (IL-1*β*, IL-6, and TNF-*α*, *n* = 3) vs. NC,  ^*∗∗∗*^*P<*0.001.

Then, we aimed to verify the molecular mechanism by which Sestrin2 and NRF2 affect ER stress-dependent ferroptosis. First, the binding relationship between Sestrin2 and NRF2 was verified by immunoprecipitation (Figure 4(a)). The results showed that knockdown of NRF2 increased the expression levels of GRP78, CHOP, XBP-1, HO-1, *α*-SMA, Collagen-I, Collagen-III, FN1, and inflammation-related factors (IL-1*β*, IL-6, and TNF-*α*), and the levels of ROS, Fe^2+^, and MDA. However, the expression levels of GPX4, FTH1, *E*-cadherin, and SOD were decreased. Furthermore, overexpression of Sestrin2 after knockdown of NRF2 showed no significant effect on these indices (Figure 4(b)–4(i)). The results show that Sestrin2 targets NRF2 and that NRF2 is a downstream signal of Sestrin2. Sestrin2 regulates the NRF2/ATF4 pathway, thereby affecting the level of ER stress-dependent ferroptosis.

### 3.5. The Effect of Sestrin2 on ER Stress-Dependent Ferroptosis

**Figure 5:**
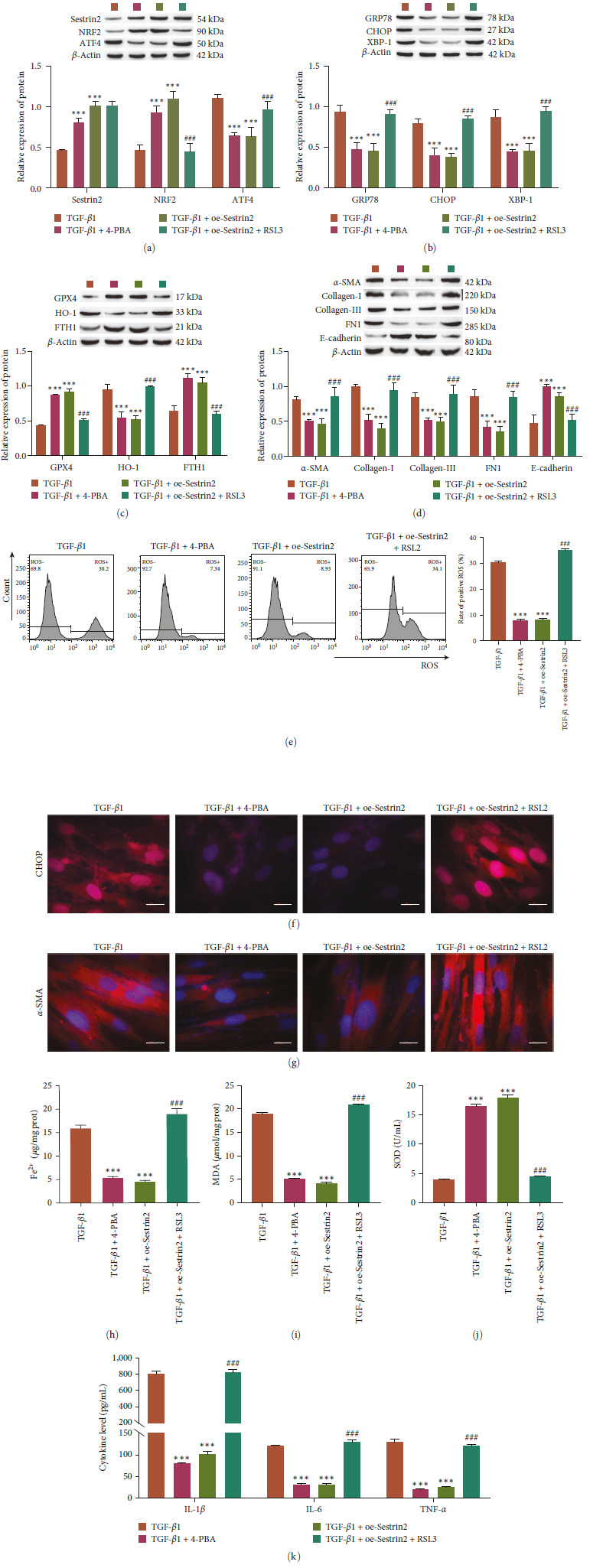
A ferroptosis activator reverses the effect of Sestrin2 on pulmonary fibrosis. (a) Western blot analysis of the expression of Sestrin2, NRF2, and ATF4 (*n* = 3). (b) Western blot analysis of the expression of ER stress-related proteins (GRP78, CHOP, and XBP-1, *n* = 3). (c) Western blot analysis of the expression of ferroptosis-related proteins (GPX4, HO-1, and FTH1, *n* = 3). (d) Western blot analysis of the expression of FMT-related proteins (*α*-SMA, collagen-I, collagen-III, FN1, and E-cadherin, *n* = 3). (e) Flow cytometric analysis of ROS levels. (f and g) Immunofluorescence analysis of CHOP and *α*-SMA expression (scale bar = 20 *µ*m, *n* = 3). (h) Analysis of Fe^2+^ levels. (i, j) Oxidative stress factor analysis to determine the expression of MDA and SOD (*n* = 3). (k) ELISA analysis of the expression of inflammation-related factors (IL-1*β*, IL-6, and TNF-*α*, *n* = 3) vs. TGF-*β*1,  ^*∗∗∗*^*P<*0.001 vs. TGF-*β*1 + oe-Sestrin2, ^###^*P<*0.001.

To further verify the effect of Sestrin2 on ER stress and ferroptosis in PF cells, an ER stress inhibitor (4-PBA) and a ferroptosis inducer (RSL3) were used. Compared with that in the TGF-*β*1 group, Sestrin2 overexpression or 4-PBA treatment inhibited the expression of ATF4 and promoted the expression of Sestrin2 and NRF2. Compared with those in the TGF-*β*1+oe-Sestrin2 group, RSL3 increased ATF4 levels and inhibited NRF2 levels (Figure 5(a)). Compared with that in the TGF-*β*1 group, Sestrin2 overexpression or 4-PBA inhibited the expression of the proteins GRP78, CHOP, XBP-1, HO-1, *α*-SMA, Collagen-I, Collagen-III, and FN1 and promoted the expression of the proteins GPX4, FTH1, and *E*-cadherin. Compared with that in the TGF-*β*1 + oe-Sestrin2 group, RSL3 increased the protein levels of GRP78, CHOP, XBP-1, HO-1, *α*-SMA, Collagen-I, Collagen-III, and FN1 and inhibited the protein levels of GPX4, FTH1, and *E*-cadherin (Figure 5(b)–5(d)). The results of immunofluorescence analysis of CHOP and *α*-SMA were consistent with the western blot results (Figures 5(f) and 5(g)). In addition, Sestrin2 overexpression or 4-PBA inhibited the production of ROS and the accumulation of Fe^2+^ and MDA and promoted the expression of SOD compared with those in the TGF-*β*1 group. Compared with those in the TGF-*β*1 + oe-Sestrin2 group, RSL3 reversed these results (Figure 5(e) and 5(h)–5(j). In addition, ELISA showed that Sestrin2 overexpression or 4-PBA treatment decreased inflammatory factors (IL-1*β*, IL-6, and TNF-*α*) (Figure 5(k)).

### 3.6. Sestrin2 Alleviates Pulmonary Fibrosis by Reducing ER Stress-Dependent Ferroptosis via the NRF2/ATF4 Pathway

**Figure 6:**
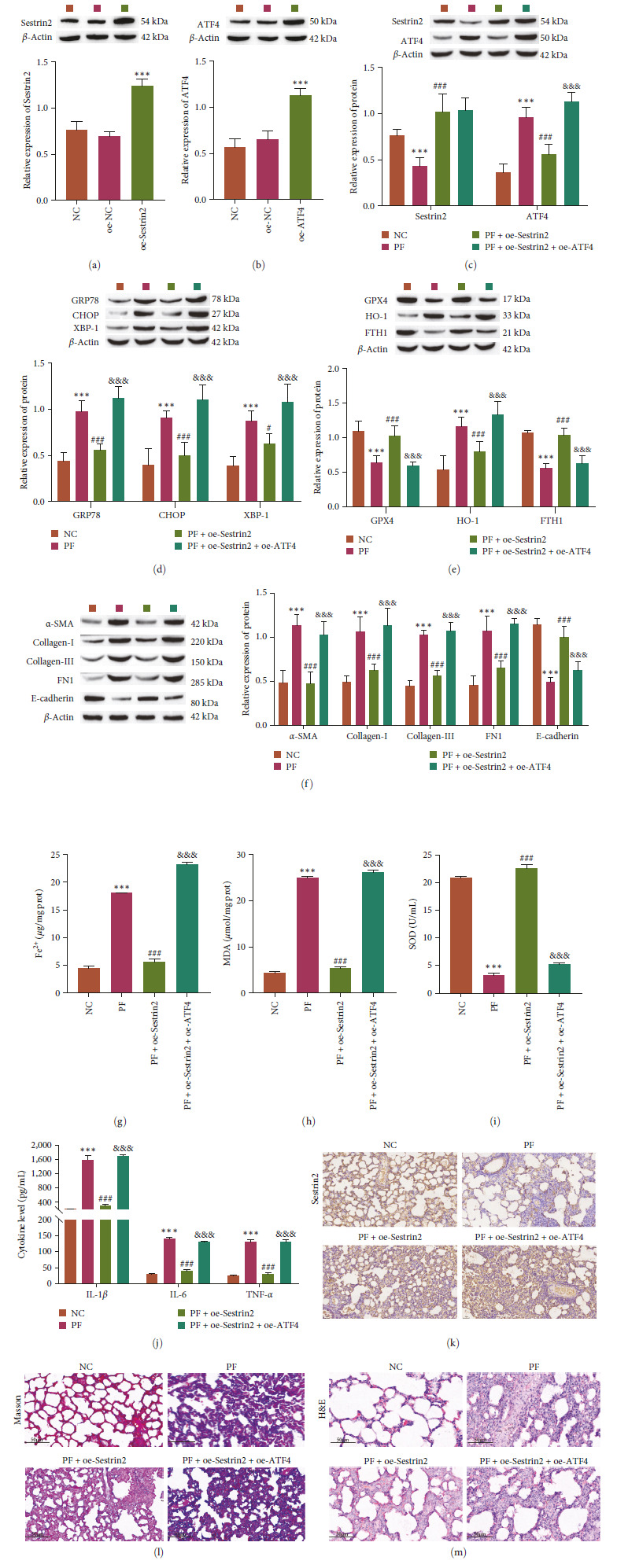
Sestrin2 alleviates endoplasmic reticulum stress-dependent ferroptosis through the NRF2/ATF4 pathway to reduce pulmonary fibrosis in vivo. (a–c) Western blot analysis of the expression of NRF2 and ATF4 (*n* = 5). (d) Western blot analysis of the expression of ER stress-related proteins (GRP78, CHOP, and XBP-1, *n* = 5). (e) Western blot analysis of the expression of ferroptosis-related proteins (GPX4, HO-1, and FTH1, *n* = 5). (f) Western blot analysis of the expression of FMT-related proteins (*α*-SMA, collagen-I, collagen-III, FN1, and *E*-cadherin, *n* = 5). (g) Analysis of Fe^2+^ level (*n* = 5). (h and i) Oxidative stress factor analysis to determine the expression of MDA and SOD (*n* = 5). (j) ELISA analysis of the expression of inflammation-related factors (IL-1*β*, IL-6, and TNF-*α*, *n* = 5). (k) Immunohistochemical staining to observe the expression of Sestrin2 (scale bar = 100 *µ*m, *n* = 5). (l) Masson staining to observe the morphology associated with pulmonary fibrosis (scale bar = 100 *µ*m, *n* = 5). (m) HE staining to observe the morphology associated with pulmonary fibrosis (scale bar = 100 *µ*m, *n* = 5) vs. NC,  ^*∗∗∗*^*P<*0.001 vs. PF, ^#^*P<*0.05, ^###^*P<*0.001 vs. PF + oe-Sestrin2, ^&&&^*P<*0.001.

To further verify that the effect of Sestrin2 on PF involved reducing endoplasmic reticulum stress-dependent ferroptosis through the NRF2/ATF4 pathway, we examined the effect of Sestrin2 on PF in vivo. Western blot analysis showed successful overexpression of Sestrin2 and ATF4 (Figures 6(a) and 6(b)). Moreover, the results showed that the expression of Sestrin2 was inhibited and the expression of ATF4 was increased in the lung tissue of mice with PF (Figure 6(c)). After Sestrin2 overexpression, the level of ER stress decreased, and ferroptosis was inhibited. Furthermore, Sestrin2 overexpression inhibited the transformation of fibroblasts into myofibroblasts. Further overexpression of ATF4 reversed these effects (Figure 6(d)–6(f)). Compared with those in mice with PF, Sestrin2 overexpression decreased the accumulation of Fe^2+^ and oxidative stress levels in lung tissue, and inflammation was inhibited to some extent (Figure 6(g)–6(j)). Immunohistochemical analysis showed that the expression of Sestrin2 was inhibited in the lung tissue of the mice with PF (Figure 6(k)). In addition, collagen fibers were deposited, the alveolar space was obviously widened, and alveolar structure was disordered, collapsed or fuzed in the lung tissue of mice with PF. After Sestrin2 overexpression, the damage to collagen fibers and structure in the lung tissue of mice was significantly alleviated (Figures 6(l) and 6(m)). These results suggest that sestrin2 can improve the pathological injury of PF in mice.

## 4. Discussion

PF, especially IPF, is a chronic progressive disease with high mortality and limited treatment options in which innate and adaptive inflammatory processes are involved [27]. The occurrence and development of PF involve many kinds of immune cells, including neutrophil, macrophage, mast cells, T cells, and B cells [3, 28]. In this work, it was found abnormal expression of TNF-*α*, IL-6, and IL-1*β* in TGF-*β*1-induced cell model and bleomycin-induced mice, which may be closely related to the development of PF. I will explore the relationship between PF and immune organs, immune cells and immunocompetent substances in further studies.

Sestrin2 is a significantly conserved stress-inducible metabolic protein involved in DNA damage, apoptosis, autophagy, endoplasmic reticulum stress, oxidative stress, and fibrogenesis that maintains metabolic homeostasis and may serve as a late-model biomarker and therapeutic target for the various diseases [29]. Early studies have demonstrated the irreplaceable role of Sestrin2 in the progression of fibrotic diseases; for instance, Sestrin2 inhibits hepatic stellate cell activation and arrests TGF-*β*1-mediated liver fibrosis [30, 31]. Sestrin2 can improve hepatic fibrosis by inhibiting activation of the AMPK signaling pathway and mTORC1/S6/4E-BP signaling pathway [32]. Sestrin2 downregulation in rat kidneys was accompanied by glomerulosclerosis and severe periglomerular fibrosis [33]. Recently, Yang et al. [9] suggested that Sestrin2 was involved in regulating PF through a ceRNA mechanism. However, the mechanisms by which Sestrin2 regulate PF need to be further investigated. We found that FMT was inhibited in MRC-5 cells, inflammation levels were reduced and cell proliferation viability was increased after Sestrin2 overexpression. Therefore, we hypothesized that Sestrin2 could alleviate the development of PF and further investigated the molecular mechanism of this alleviation.

ER stress induces excessive ROS production; the accumulation of ROS in turn accelerates protein aggregation and ER stress [34, 35], and ROS-induced lipid peroxidation plays a key role in cellular ferroptosis [36, 37]. This study showed that under ER stress conditions, ROS accumulation accelerated protein aggregation and ER stress, and ROS-induced lipid peroxidation played a key role in cellular ferroptosis, thereby promoting disease development. Under ER stress conditions, Sestrin2 can be upregulated to inhibit ER stress-mediated apoptosis [38]. Sestrin2 was shown to prevent protein overproduction, reduce the sensitivity of cells to ER stress and slow protein responses [38]. It was also shown that ER stress conditions activate ATF4 and NRF2 to regulate cellular oxidative stress and ferroptosis [39–43]. Consistent with the earlier studies, we found that overexpression of Sestrin2-inhibited ROS production and the level of TNF-*α*, IL-6, and IL-1*β* to attenuate endoplasmic reticulum stress and ferroptosis, thereby ameliorating lung fibrosis. In addition, we observed that NRF2/ATF4 activation alleviated endoplasmic reticulum stress-dependent ferroptosis to attenuate PF.

Previous studies have shown that the upregulation of Sestrin2 is triggered by the transcription factors ATF4 and NRF2 [13]. Sestrin2 protects dendritic cells from sepsis-induced ferroptosis and may act as an antioxidant by downregulating the ATF4/CHOP/CHAC1 signaling pathway [44]. Sestrin2 is involved in the NRF2-regulated antioxidant pathway [45]. We also demonstrated an interaction between Sestrin2 and NRF2 by immunoprecipitation. In addition, we found that Sestrin2 inhibited the expression of ATF4 and increased the expression of NRF2. The effect of Sestrin2 was reversed by the overexpression of ATF4. These results suggest that the role of Sestrin2 in alleviating endoplasmic reticulum stress-dependent ferroptosis levels to attenuate PF is related to NRF2/ATF4 signaling.

Ferroptosis plays a key role in the development of PF, and excess iron can lead to PF, which is correlated with increased lipid peroxidation and reduced GPX4 activity in lung tissue [46]. Ferroptosis can be regulated by the immune cells, but the specific mechanism is still unclear [17]. Recent preclinical studies suggest that ferroptosis may be a therapeutic target for fibrotic diseases, such as liver fibrosis, PF, renal fibrosis, and myocardial fibrosis [47], and the modulation of cellular ferroptosis could be a therapeutic strategy. In the present study, we found that the ferroptosis inducer RSL3 reversed the ameliorative effect of Sestrin2 on PF at the cellular level, demonstrating that Sestrin2 attenuates PF by alleviating endoplasmic reticulum stress-dependent ferroptosis. In addition, we also demonstrated that Sestrin2 attenuates PF by alleviating endoplasmic reticulum stress-dependent ferroptosis levels via the NRF2/ATF4 pathway, which inhibits lung tissue structural lesions and collagen fiber accumulation at the animal level. These findings suggest that Sestrin2 may be a new target for the clinical treatment of PF.

## 5. Conclusion

In conclusion, our study confirmed that Sestrin2 alleviated inflammation and endoplasmic reticulum stress-dependent ferroptosis through the NRF2/ATF4 pathway to reduce PF at the cellular and animal levels, which may indicate new therapeutic targets and ideas for the clinical treatment of PF. However, the limitation of our study is that only one molecular pathway (NRF2/ATF4) was explored, and Sestrin2 may also alleviate PF through other pathways and immune ways, which will be explored in the follow-up studies.

## Figures and Tables

**Table 1:** Primer sequences.

Primer	Sequence (5′–3′)
Sestrin2-F	CTACCCTGAGAAGACGACC
Sestrin2-R	AAGTTCACATGAACCTTCTCTG
GAPDH-F	AGATCCCTCCAAAATCAAGTGG
GAPDH-R	GGCAGAGATGATGACCCTTTT

## Data Availability

The datasets used and/or analyzed during the current study are available from the corresponding author upon reasonable request.
